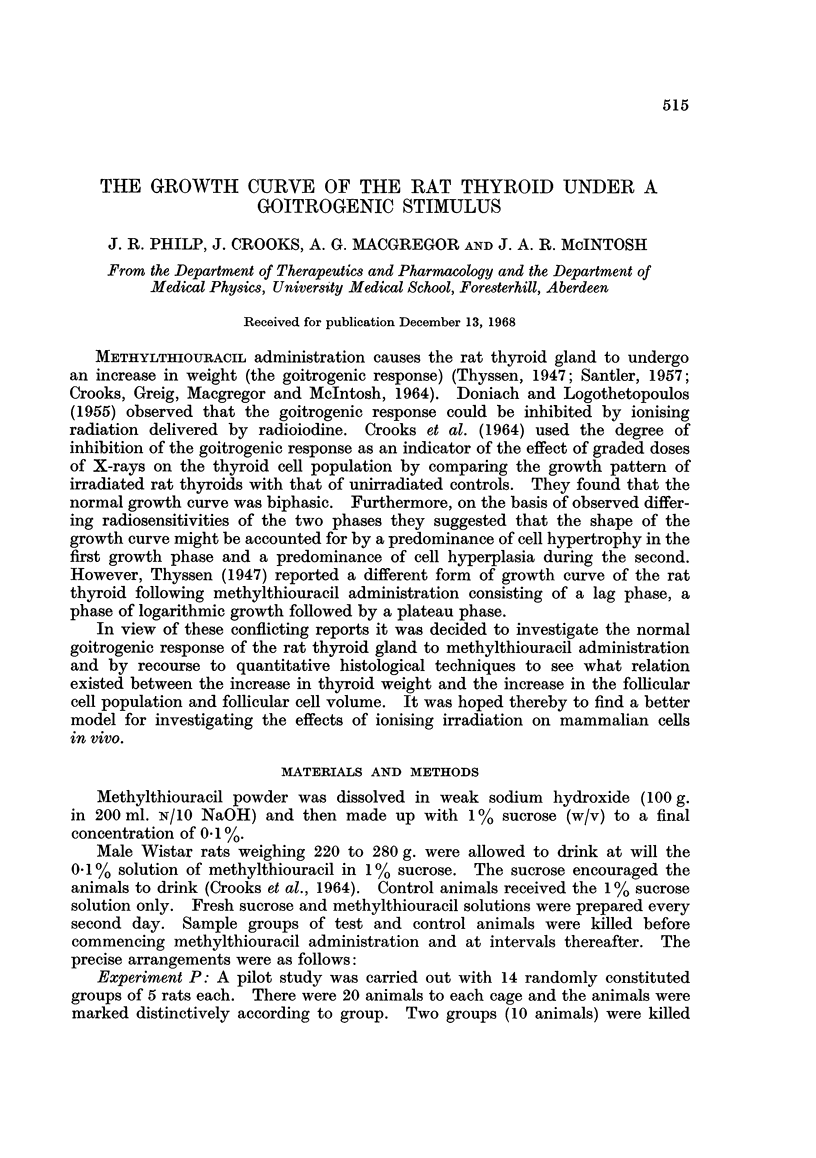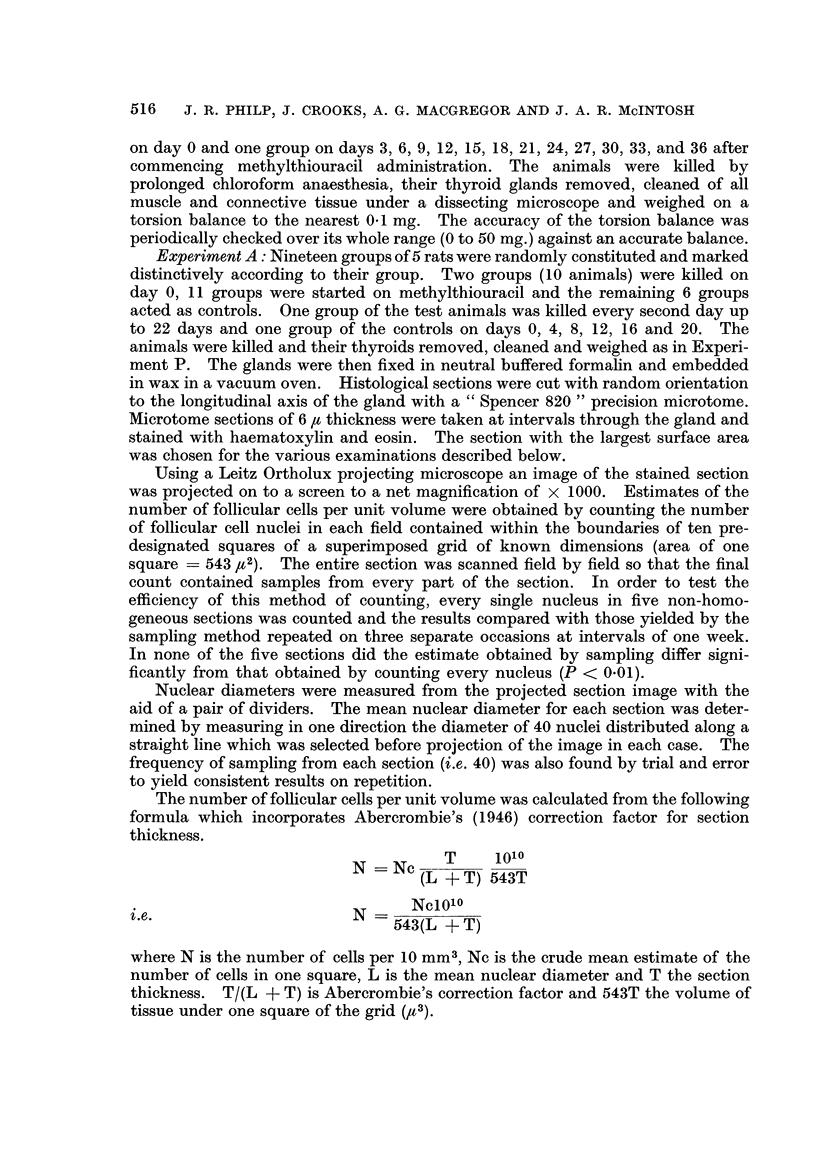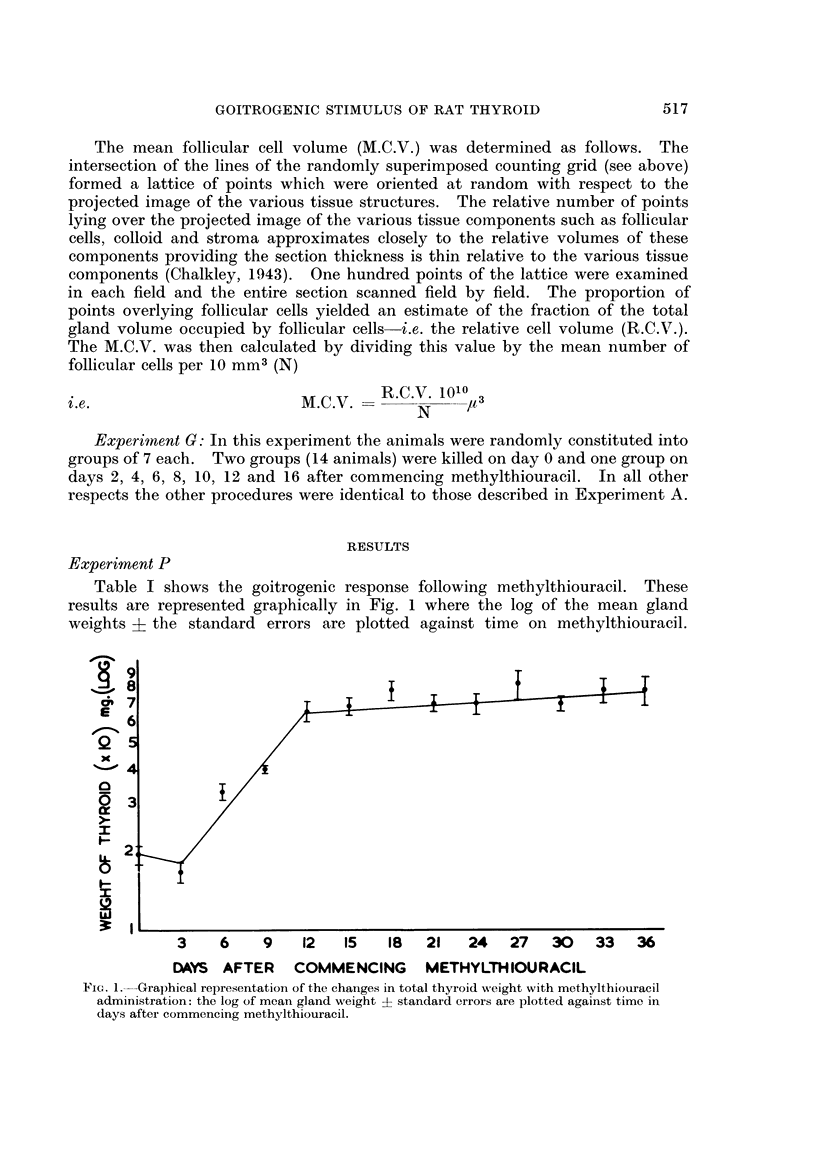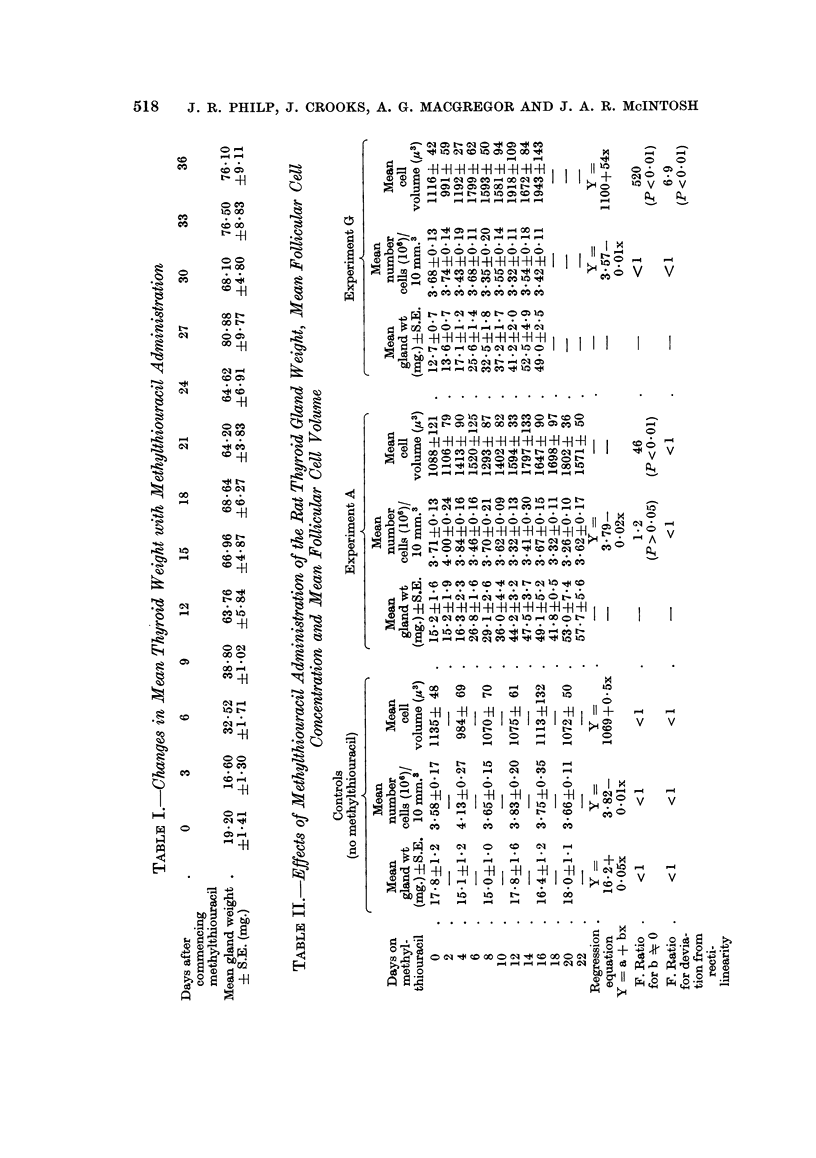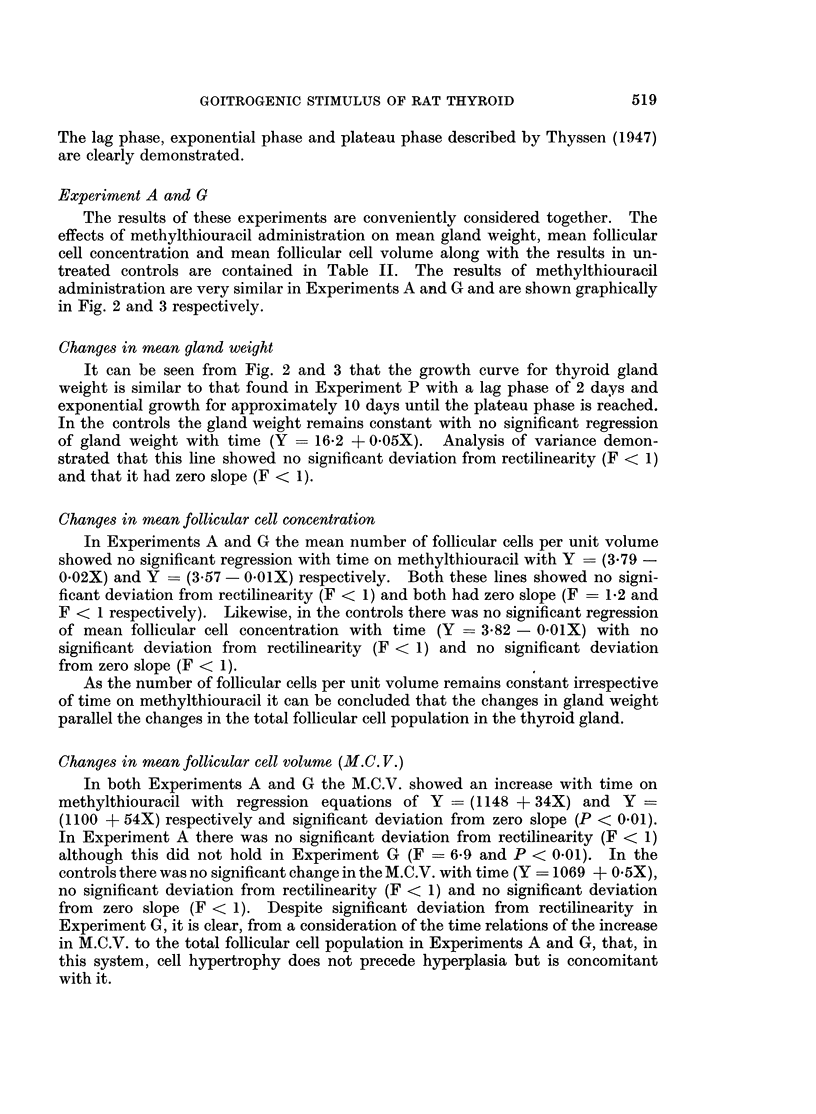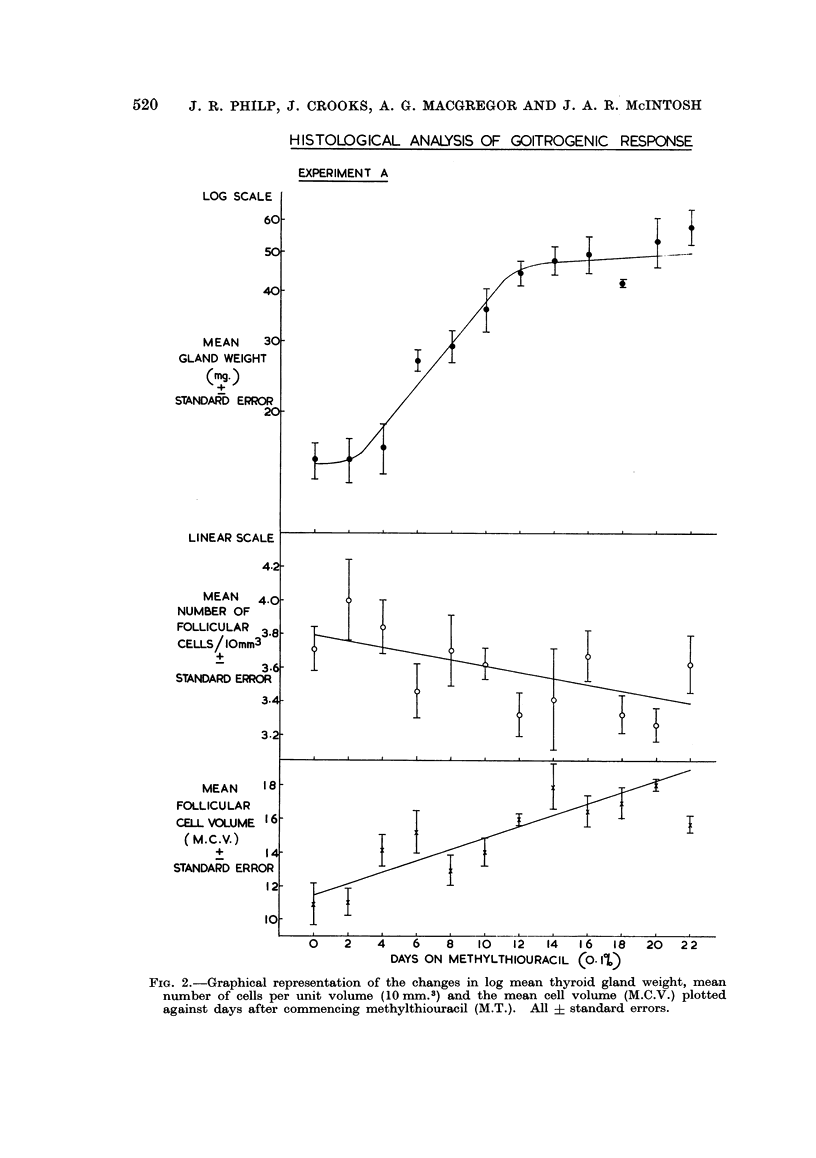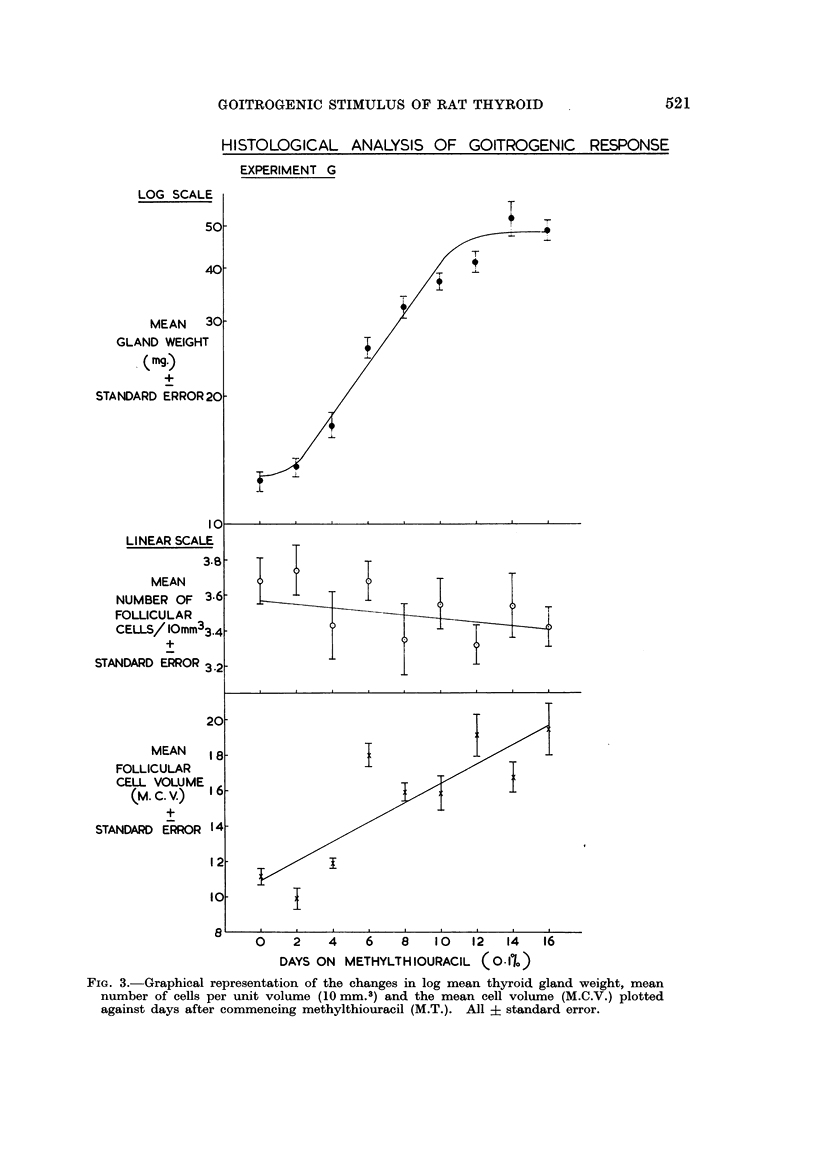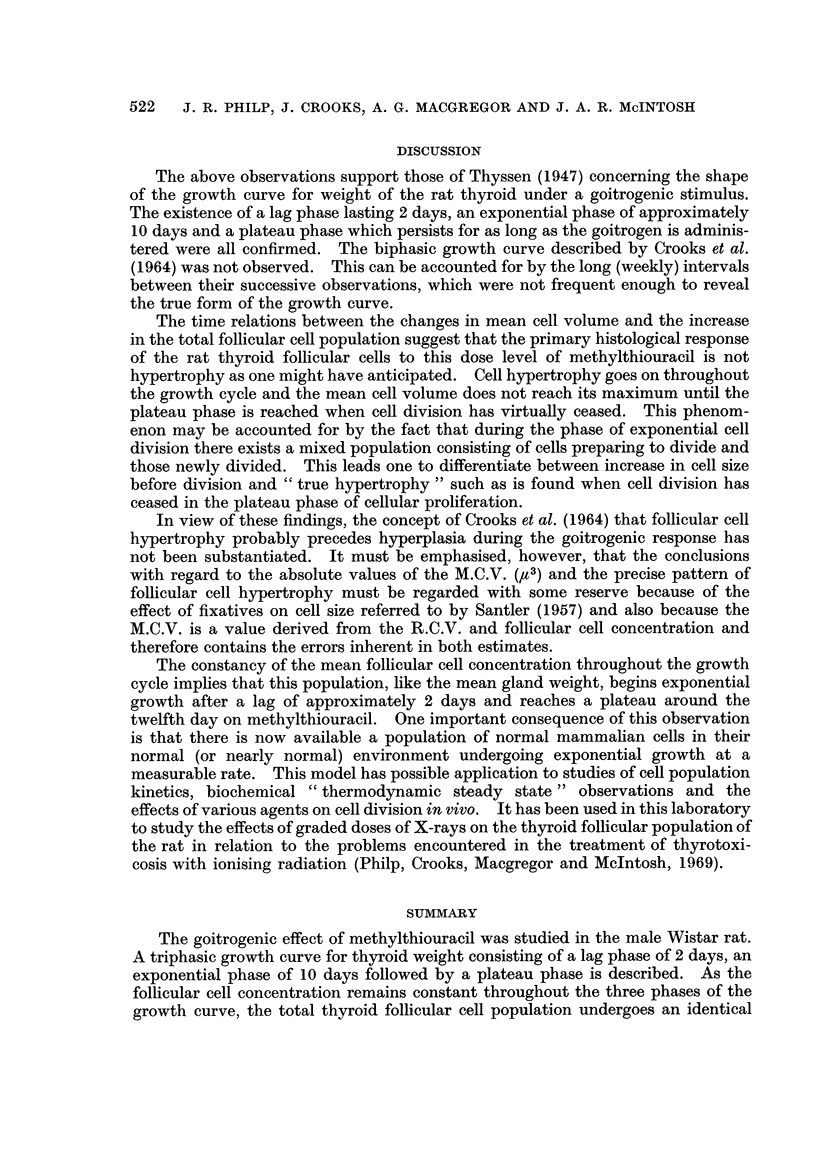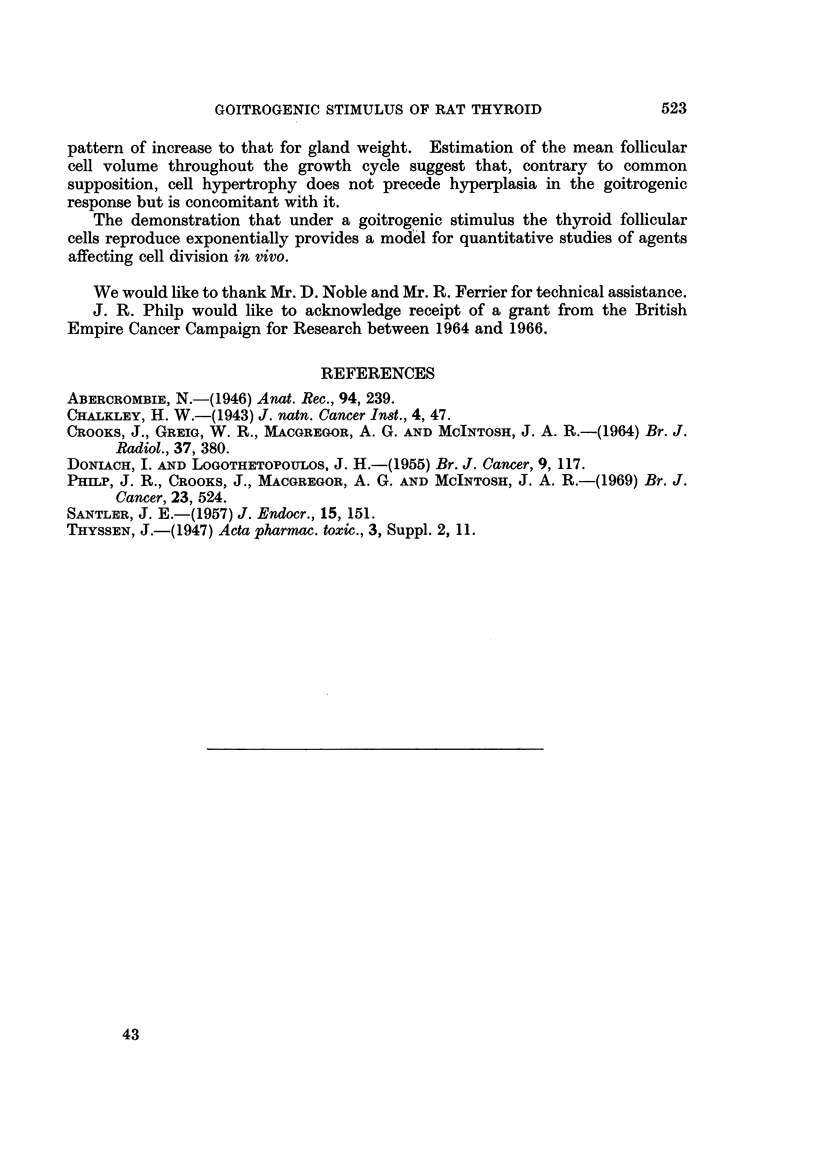# The growth curve of the rat thyroid under a goitrogenic stimulus.

**DOI:** 10.1038/bjc.1969.64

**Published:** 1969-09

**Authors:** J. R. Philp, J. Crooks, A. G. Macgregor, J. A. McIntosh


### THE GROWTH CURVE OF THE RAT THYROID UNDER A GOITROGENIC STIMULUS

J. R. PHILP, J. CROOKS, A. G. MACGREGOR AND J. A. R. McINTOSH

*From the Department of Therapeutics and Pharmacology and the Department of Medical Physics, University Medical School, Foresterhill, Aberdeen*

Received for publication December 13, 1968

METHYLTHIOURACIL administration causes the rat thyroid gland to undergo an increase in weight (the goitrogenic response) (Thyssen, 1947; Santler, 1957; Crooks, Greig, Macgregor and McIntosh, 1964). Doniach and Logothetopoulos (1955) observed that the goitrogenic response could be inhibited by ionising radiation delivered by radioiodine. Crooks *et al.* (1964) used the degree of inhibition of the goitrogenic response as an indicator of the effect of graded doses of X-rays on the thyroid cell population by comparing the growth pattern of irradiated rat thyroids with that of unirradiated controls. They found that the normal growth curve was biphasic. Furthermore, on the basis of observed differing radiosensitivities of the two phases they suggested that the shape of the growth curve might be accounted for by a predominance of cell hypertrophy in the first growth phase and a predominance of cell hyperplasia during the second. However, Thyssen (1947) reported a different form of growth curve of the rat thyroid following methylthiouracil administration consisting of a lag phase, a phase of logarithmic growth followed by a plateau phase.

In view of these conflicting reports it was decided to investigate the normal goitrogenic response of the rat thyroid gland to methylthiouracil administration and by recourse to quantitative histological techniques to see what relation existed between the increase in thyroid weight and the increase in the follicular cell population and follicular cell volume. It was hoped thereby to find a better model for investigating the effects of ionising irradiation on mammalian cells *in vivo.*

### MATERIALS AND METHODS

Methylthiouracil powder was dissolved in weak sodium hydroxide (100 g. in 200 ml. N/10 NaOH) and then made up with 1% sucrose (w/v) to a final concentration of 0·1%.

Male Wistar rats weighing 220 to 280 g. were allowed to drink at will the 0·1% solution of methylthiouracil in 1% sucrose. The sucrose encouraged the animals to drink (Crooks *et al.*, 1964). Control animals received the 1% sucrose solution only. Fresh sucrose and methylthiouracil solutions were prepared every second day. Sample groups of test and control animals were killed before commencing methylthiouracil administration and at intervals thereafter. The precise arrangements were as follows:

*Experiment P:* A pilot study was carried out with 14 randomly constituted groups of 5 rats each. There were 20 animals to each cage and the animals were marked distinctively according to group. Two groups (10 animals) were killed on day 0 and one group on days 3, 6, 9, 12, 15, 18, 21, 24, 27, 30, 33, and 36 after commencing methylthiouracil administration. The animals were killed by prolonged chloroform anaesthesia, their thyroid glands removed, cleaned of all muscle and connective tissue under a dissecting microscope and weighed on a torsion balance to the nearest 0·1 mg. The accuracy of the torsion balance was periodically checked over its whole range (0 to 50 mg.) against an accurate balance.

*Experiment A :* Nineteen groups of 5 rats were randomly constituted and marked distinctively according to their group. Two groups (10 animals) were killed on day 0, 11 groups were started on methylthiouracil and the remaining 6 groups acted as controls. One group of the test animals was killed every second day up to 22 days and one group of the controls on days 0, 4, 8, 12, 16 and 20. The animals were killed and their thyroids removed, cleaned and weighed as in Experiment P. The glands were then fixed in neutral buffered formalin and embedded in wax in a vacuum oven. Histological sections were cut with random orientation to the longitudinal axis of the gland with a " Spencer 820 " precision microtome. Microtome sections of 6 $\mu$ thickness were taken at intervals through the gland and stained with haematoxylin and eosin. The section with the largest surface area was chosen for the various examinations described below.

Using a Leitz Ortholux projecting microscope an image of the stained section was projected on to a screen to a net magnification of $\times$ 1000. Estimates of the number of follicular cells per unit volume were obtained by counting the number of follicular cell nuclei in each field contained within the boundaries of ten predesignated squares of a superimposed grid of known dimensions (area of one square = 543 $\mu^2$). The entire section was scanned field by field so that the final count contained samples from every part of the section. In order to test the efficiency of this method of counting, every single nucleus in five non-homogeneous sections was counted and the results compared with those yielded by the sampling method repeated on three separate occasions at intervals of one week. In none of the five sections did the estimate obtained by sampling differ significantly from that obtained by counting every nucleus ($P < 0{\cdot}01$).

Nuclear diameters were measured from the projected section image with the aid of a pair of dividers. The mean nuclear diameter for each section was determined by measuring in one direction the diameter of 40 nuclei distributed along a straight line which was selected before projection of the image in each case. The frequency of sampling from each section (*i.e.* 40) was also found by trial and error to yield consistent results on repetition.

The number of follicular cells per unit volume was calculated from the following formula which incorporates Abercrombie's (1946) correction factor for section thickness.

$$N = Nc \frac{T}{(L + T)} \frac{10^{10}}{543T}$$

*i.e.*

$$N = \frac{Nc10^{10}}{543(L + T)}$$

where N is the number of cells per 10 mm$^3$, Nc is the crude mean estimate of the number of cells in one square, L is the mean nuclear diameter and T the section thickness. T/(L + T) is Abercrombie's correction factor and 543T the volume of tissue under one square of the grid ($\mu^3$).

The mean follicular cell volume (M.C.V.) was determined as follows. The intersection of the lines of the randomly superimposed counting grid (see above) formed a lattice of points which were oriented at random with respect to the projected image of the various tissue structures. The relative number of points lying over the projected image of the various tissue components such as follicular cells, colloid and stroma approximates closely to the relative volumes of these components providing the section thickness is thin relative to the various tissue components (Chalkley, 1943). One hundred points of the lattice were examined in each field and the entire section scanned field by field. The proportion of points overlying follicular cells yielded an estimate of the fraction of the total gland volume occupied by follicular cells—*i.e.* the relative cell volume (R.C.V.). The M.C.V. was then calculated by dividing this value by the mean number of follicular cells per 10 $mm^3$ (N)

*i.e.* $$\text{M.C.V.} = \frac{\text{R.C.V.}\ 10^{10}}{\text{N}}\mu^3$$

*Experiment G:* In this experiment the animals were randomly constituted into groups of 7 each. Two groups (14 animals) were killed on day 0 and one group on days 2, 4, 6, 8, 10, 12 and 16 after commencing methylthiouracil. In all other respects the other procedures were identical to those described in Experiment A.

### RESULTS

### *Experiment P*

Table I shows the goitrogenic response following methylthiouracil. These results are represented graphically in Fig. 1 where the log of the mean gland weights ± the standard errors are plotted against time on methylthiouracil.

FIG. 1.—Graphical representation of the changes in total thyroid weight with methylthiouracil administration: the log of mean gland weight ± standard errors are plotted against time in days after commencing methylthiouracil.

TABLE I.—*Changes in Mean Thyroid Weight with Methylthiouracil Administration*

| Days after commencing methylthiouracil | 0 | 3 | 6 | 9 | 12 | 15 | 18 | 21 | 24 | 27 | 30 | 33 | 36 |
|---|---|---|---|---|---|---|---|---|---|---|---|---|---|
| Mean gland weight ± S.E. (mg.) | 19·20 ±1·41 | 16·60 ±1·30 | 32·52 ±1·71 | 38·80 ±1·02 | 63·76 ±5·84 | 66·96 ±4·87 | 68·64 ±6·27 | 64·20 ±3·83 | 64·62 ±6·91 | 80·88 ±9·77 | 68·10 ±4·80 | 76·50 ±8·83 | 76·10 ±9·11 |

TABLE II.—*Effects of Methylthiouracil Administration of the Rat Thyroid Gland Weight, Mean Follicular Cell Concentration and Mean Follicular Cell Volume*

| Days on methyl-thiouracil | Controls (no methylthiouracil) Mean gland wt (mg.) ±S.E. | Controls Mean number cells ($10^6$)/10 mm.$^3$ | Controls Mean cell volume ($\mu^3$) | Experiment A Mean gland wt (mg.) ±S.E. | Experiment A Mean number cells ($10^6$)/10 mm.$^3$ | Experiment A Mean cell volume ($\mu^3$) | Experiment G Mean gland wt (mg.) ±S.E. | Experiment G Mean number cells ($10^6$)/10 mm.$^3$ | Experiment G Mean cell volume ($\mu^3$) |
|---|---|---|---|---|---|---|---|---|---|
| 0 | 17·8±1·2 | 3·58±0·17 | 1135± 48 | 15·2±1·6 | 3·71±0·13 | 1088±121 | 12·7±0·7 | 3·68±0·13 | 1116± 42 |
| 2 | — | — | — | 15·2±1·9 | 4·00±0·24 | 1106± 79 | 13·6±0·7 | 3·74±0·14 | 991± 59 |
| 4 | 15·1±1·2 | 4·13±0·27 | 984± 69 | 16·3±2·3 | 3·84±0·16 | 1413± 90 | 17·1±1·2 | 3·43±0·19 | 1192± 27 |
| 6 | — | — | — | 26·8±1·6 | 3·46±0·16 | 1520±125 | 25·6±1·4 | 3·68±0·11 | 1799± 62 |
| 8 | 15·0±1·0 | 3·65±0·15 | 1070± 70 | 29·1±2·6 | 3·70±0·21 | 1293± 87 | 32·5±1·8 | 3·35±0·20 | 1593± 50 |
| 10 | — | — | — | 36·0±4·4 | 3·62±0·09 | 1402± 82 | 37·2±1·7 | 3·55±0·14 | 1581± 94 |
| 12 | 17·8±1·6 | 3·83±0·20 | 1075± 61 | 44·2±3·2 | 3·32±0·13 | 1594± 33 | 41·2±2·0 | 3·32±0·11 | 1918±109 |
| 14 | — | — | — | 47·5±3·7 | 3·41±0·30 | 1797±133 | 52·5±4·9 | 3·54±0·18 | 1672± 84 |
| 16 | 16·4±1·2 | 3·75±0·35 | 1113±132 | 49·1±5·2 | 3·67±0·15 | 1647± 90 | 49·0±2·5 | 3·42±0·11 | 1943±143 |
| 18 | — | — | — | 41·8±0·5 | 3·32±0·11 | 1698± 97 | — | — | — |
| 20 | 18·0±1·1 | 3·66±0·11 | 1072± 50 | 53·0±7·4 | 3·26±0·10 | 1802± 36 | — | — | — |
| 22 | — | — | — | 57·7±5·6 | 3·62±0·17 | 1571± 50 | — | — | — |
| Regression equation Y = a + bx | Y = 16·2+ 0·05x | Y = 3·82− 0·01x | Y = 1069+0·5x | — | Y = 3·79− 0·02x | — | — | Y = 3·57− 0·01x | Y = 1100+54x |
| F. Ratio for b ≠ 0 | <1 | <1 | <1 | — | 1·2 ($P>0·05$) | 46 ($P<0·01$) | — | <1 | 520 ($P<0·01$) |
| F. Ratio for deviation from rectilinearity | <1 | <1 | <1 | — | <1 | <1 | — | <1 | 6·9 ($P<0·01$) |

The lag phase, exponential phase and plateau phase described by Thyssen (1947) are clearly demonstrated.

*Experiment A and G*

The results of these experiments are conveniently considered together. The effects of methylthiouracil administration on mean gland weight, mean follicular cell concentration and mean follicular cell volume along with the results in untreated controls are contained in Table II. The results of methylthiouracil administration are very similar in Experiments A and G and are shown graphically in Fig. 2 and 3 respectively.

*Changes in mean gland weight*

It can be seen from Fig. 2 and 3 that the growth curve for thyroid gland weight is similar to that found in Experiment P with a lag phase of 2 days and exponential growth for approximately 10 days until the plateau phase is reached. In the controls the gland weight remains constant with no significant regression of gland weight with time ($Y = 16{\cdot}2 + 0{\cdot}05X$). Analysis of variance demonstrated that this line showed no significant deviation from rectilinearity ($F < 1$) and that it had zero slope ($F < 1$).

*Changes in mean follicular cell concentration*

In Experiments A and G the mean number of follicular cells per unit volume showed no significant regression with time on methylthiouracil with $Y = (3{\cdot}79 - 0{\cdot}02X)$ and $Y = (3{\cdot}57 - 0{\cdot}01X)$ respectively. Both these lines showed no significant deviation from rectilinearity ($F < 1$) and both had zero slope ($F = 1{\cdot}2$ and $F < 1$ respectively). Likewise, in the controls there was no significant regression of mean follicular cell concentration with time ($Y = 3{\cdot}82 - 0{\cdot}01X$) with no significant deviation from rectilinearity ($F < 1$) and no significant deviation from zero slope ($F < 1$).

As the number of follicular cells per unit volume remains constant irrespective of time on methylthiouracil it can be concluded that the changes in gland weight parallel the changes in the total follicular cell population in the thyroid gland.

*Changes in mean follicular cell volume (M.C.V.)*

In both Experiments A and G the M.C.V. showed an increase with time on methylthiouracil with regression equations of $Y = (1148 + 34X)$ and $Y = (1100 + 54X)$ respectively and significant deviation from zero slope ($P < 0{\cdot}01$). In Experiment A there was no significant deviation from rectilinearity ($F < 1$) although this did not hold in Experiment G ($F = 6{\cdot}9$ and $P < 0{\cdot}01$). In the controls there was no significant change in the M.C.V. with time ($Y = 1069 + 0{\cdot}5X$), no significant deviation from rectilinearity ($F < 1$) and no significant deviation from zero slope ($F < 1$). Despite significant deviation from rectilinearity in Experiment G, it is clear, from a consideration of the time relations of the increase in M.C.V. to the total follicular cell population in Experiments A and G, that, in this system, cell hypertrophy does not precede hyperplasia but is concomitant with it.

Fig. 2.—Graphical representation of the changes in log mean thyroid gland weight, mean number of cells per unit volume (10 mm.$^3$) and the mean cell volume (M.C.V.) plotted against days after commencing methylthiouracil (M.T.). All ± standard errors.

FIG. 3.—Graphical representation of the changes in log mean thyroid gland weight, mean number of cells per unit volume (10 mm.$^3$) and the mean cell volume (M.C.V.) plotted against days after commencing methylthiouracil (M.T.). All ± standard error.

### DISCUSSION

The above observations support those of Thyssen (1947) concerning the shape of the growth curve for weight of the rat thyroid under a goitrogenic stimulus. The existence of a lag phase lasting 2 days, an exponential phase of approximately 10 days and a plateau phase which persists for as long as the goitrogen is administered were all confirmed. The biphasic growth curve described by Crooks *et al.* (1964) was not observed. This can be accounted for by the long (weekly) intervals between their successive observations, which were not frequent enough to reveal the true form of the growth curve.

The time relations between the changes in mean cell volume and the increase in the total follicular cell population suggest that the primary histological response of the rat thyroid follicular cells to this dose level of methylthiouracil is not hypertrophy as one might have anticipated. Cell hypertrophy goes on throughout the growth cycle and the mean cell volume does not reach its maximum until the plateau phase is reached when cell division has virtually ceased. This phenomenon may be accounted for by the fact that during the phase of exponential cell division there exists a mixed population consisting of cells preparing to divide and those newly divided. This leads one to differentiate between increase in cell size before division and " true hypertrophy " such as is found when cell division has ceased in the plateau phase of cellular proliferation.

In view of these findings, the concept of Crooks *et al.* (1964) that follicular cell hypertrophy probably precedes hyperplasia during the goitrogenic response has not been substantiated. It must be emphasised, however, that the conclusions with regard to the absolute values of the M.C.V. ($\mu^3$) and the precise pattern of follicular cell hypertrophy must be regarded with some reserve because of the effect of fixatives on cell size referred to by Santler (1957) and also because the M.C.V. is a value derived from the R.C.V. and follicular cell concentration and therefore contains the errors inherent in both estimates.

The constancy of the mean follicular cell concentration throughout the growth cycle implies that this population, like the mean gland weight, begins exponential growth after a lag of approximately 2 days and reaches a plateau around the twelfth day on methylthiouracil. One important consequence of this observation is that there is now available a population of normal mammalian cells in their normal (or nearly normal) environment undergoing exponential growth at a measurable rate. This model has possible application to studies of cell population kinetics, biochemical " thermodynamic steady state " observations and the effects of various agents on cell division *in vivo*. It has been used in this laboratory to study the effects of graded doses of X-rays on the thyroid follicular population of the rat in relation to the problems encountered in the treatment of thyrotoxicosis with ionising radiation (Philp, Crooks, Macgregor and McIntosh, 1969).

### SUMMARY

The goitrogenic effect of methylthiouracil was studied in the male Wistar rat. A triphasic growth curve for thyroid weight consisting of a lag phase of 2 days, an exponential phase of 10 days followed by a plateau phase is described. As the follicular cell concentration remains constant throughout the three phases of the growth curve, the total thyroid follicular cell population undergoes an identical pattern of increase to that for gland weight. Estimation of the mean follicular cell volume throughout the growth cycle suggest that, contrary to common supposition, cell hypertrophy does not precede hyperplasia in the goitrogenic response but is concomitant with it.

The demonstration that under a goitrogenic stimulus the thyroid follicular cells reproduce exponentially provides a model for quantitative studies of agents affecting cell division *in vivo*.

We would like to thank Mr. D. Noble and Mr. R. Ferrier for technical assistance.

J. R. Philp would like to acknowledge receipt of a grant from the British Empire Cancer Campaign for Research between 1964 and 1966.

### REFERENCES

ABERCROMBIE, N.—(1946) *Anat. Rec.*, **94**, 239.
CHALKLEY, H. W.—(1943) *J. natn. Cancer Inst.*, **4**, 47.
CROOKS, J., GREIG, W. R., MACGREGOR, A. G. AND MCINTOSH, J. A. R.—(1964) *Br. J. Radiol.*, **37**, 380.
DONIACH, I. AND LOGOTHETOPOULOS, J. H.—(1955) *Br. J. Cancer*, **9**, 117.
PHILP, J. R., CROOKS, J., MACGREGOR, A. G. AND MCINTOSH, J. A. R.—(1969) *Br. J. Cancer*, **23**, 524.
SANTLER, J. E.—(1957) *J. Endocr.*, **15**, 151.
THYSSEN, J.—(1947) *Acta pharmac. toxic.*, **3**, Suppl. 2, 11.